# An Overall Test of Pairwise Mean Conditional Covariances in IRT

**DOI:** 10.1017/psy.2024.21

**Published:** 2025-01-03

**Authors:** Jules L. Ellis, L. Andries van der Ark, Klaas Sijtsma

**Affiliations:** 1 Faculty of Psychology, Open Universiteit, Heerlen, Netherlands; 2 Research Institute of Child Development and Education, University of Amsterdam, Amsterdam, Netherlands; 3 Department of Methodology and Statistics TSB, Tilburg University, Tilburg

**Keywords:** conditional association, monotone homogeneity model, monotone latent variable model, multidimensional measurement, unidimensional measurement

## Abstract

We study how the Conditioning on Added Regression Predictions (CARP) statistics from different item pairs can be aggregated into a single overall test of monotone homogeneity. As a pairwise statistic, we use the mean conditional covariance (MCC) or its standardized value ( 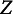 ). We use three different estimates of the covariance matrix of the pairwise test statistics: (1) the covariance matrix of the MCCs, based on the sample moments; (2) the covariance matrix of the MCCs or 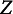 s, based on bootstrapping; and (3) the covariance matrix of the 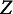 s, equated to the identity matrix. We consider various aggregation methods, including (a) the chi-bar-square statistic; (b) the preselected standardized partial sum of pairwise statistics; (c) the product of preselected 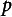 -values; (d) the minimum of preselected 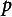 -values; and (e–h) the same statistics, but now conditioned on post-selecting only the negative values in the test sample. We study the Type 1 error rate and power of the ensuing 20 tests based on simulations. The tests with the highest power among the tests that control the Type I error rate are based on 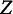 -statistics with the identity matrix: the conditional likelihood ratio test, the conditionalized product of 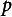 -values, the conditionalized sum of Z-values, and the preselected product of 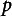 -values.

## 1 Introduction

In this paper, we develop new statistics for confirmatory tests of unidimensionality based on the nonparametric item response theory (IRT) model of monotone homogeneity (MH) (Mokken, 1971) with binary items. This model assumes that there is a unidimensional (i.e., real-valued) variable 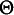 such that the items are conditionally independent given 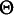 , and such that the item regressions on 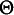 are monotone increasing. Many parametric IRT models, such as the 2PL model and the Rasch model, are a special case of MH. We develop our statistical tests for the context where researchers have the theory or hypothesis that items of a certain specified set or category all have a monotone regression on the same latent variable, while the specific shape of the regressions is unspecified; that is, it does not have to be logistic, as in the 2PL or the Rasch model, or any other function, such as the normal ogive. We assume that the objective of the test is to falsify the theory of unidimensionality. Thus, the objective is entirely aimed at fundamental theory development and not at the more pragmatic goal of building an efficient measurement tool.

Our objective of fundamental theory development rules out the possibility of assessing dimensionality with flexible parametric models such as latent class models (Douglas & Cohen, 2001; Van Onna, 2002; Vermunt, 2001) or monotone polynomial models (Falk & Cai, 2016). If such a parametric model is violated, it does not provide a convincing falsification of the theory because the violation may be caused by failure of the specific parametric assumptions. Conversely, if such a restrictive parametric model is not rejected, one may wonder whether maybe the statistical test used was not sensitive to some assumptions (e.g., van den Wollenberg, 1982).

The new test statistics will be based on the recently developed Conditioning on Added Regression Predictions (CARP) statistics of Ellis and Sijtsma (2023). The CARP testing approach is a generalization of Rosenbaum’s (1984) case 5, in which one tests nonnegativity of the covariance of an item pair, conditionally on decile groups defined by the sum score on the other items. The generalization Ellis and Sijtsma proposed using a weighted sum score instead of a simple sum score, where the weights are based on regression analysis in a training sample. Ellis and Sijtsma (2023) argued that this is currently the only known partial test of conditional association (see next section) that can detect multidimensionality within monotone IRT models. This is the reason why we focus on this test statistic.

An important limitation of the CARP test is that it pertains to a single item pair. Generally, a test has many item pairs, and it would seem logical to apply a test to each item pair, but hitherto it has not been studied how such pairwise tests can be compounded into a single test statistic. The same is true for Rosenbaum’s case 5 test. For example, if a psychological test consists of 10 items, the CARP tests would yield 45 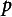 -values, one for each item pair. The main question of this article is: How can the pairwise CARP statistics be aggregated into a single omnibus test?

The next section provides some background information about the CARP tests. After the specification of the hypothesis and the relevant pairwise statistics (mean conditional covariances (MCCs) and their 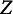 -values), we consider various methods to estimate the covariance matrix of the pairwise statistics. These estimated matrices are used in the theory of order restricted statistical inference (Robertson et al., 1988) and multiple testing (Davidov, 2011; Ellis et al., 2018) to compound them into overall statistics in 20 different ways. We will compare the mathematical structure of some of these aggregated statistics to the most prominent competitor, which is the DETECT index (Zhang & Stout, 1999a, 1999b). Next, we use Monte Carlo simulations to study the Type 1 error rates and power of the ensuing tests and select the best tests.

## 2 Conditional association and CARP tests

Rosenbaum (1984) showed that MH implies that the item score variables have the property of conditional association, which means that any two increasing functions of any subtest have a nonnegative covariance conditionally upon any function of the items that were not included in the subtest. Holland and Rosenbaum (1986) generalized this result to non-binary items. Clarke and Yuan (2001) and De Gooijer and Yuan (2011) developed statistical tests for conditional association, but it is well known that a full test of conditional association is not feasible for realistic sizes of item sets because of the large number of tested conditions and the sparseness of the relevant response patterns. Several authors have therefore focussed on what Ligtvoet (2022) recently called “partial tests of conditional association.” These tests include MTP2 (Bartolucci & Forcina, 2000; Ellis, 2015), nonnegative partial correlations (Ellis, 2014), nonnegative correlations (Mokken, 1971), and increasing item-rest regressions (“manifest monotonicity”; Junker & Sijtsma, 2000). Ellis and Sijtsma (2023) showed that all these conditions are insensitive to violations of unidimensionality if the data are generated by multiple latent variables that are independent or MTP2. They developed the CARP test, which includes Rosenbaum’s case 5 as a special case. This is currently the only known partial test of conditional association that can detect such violations. That is the reason why we focus on the CARP test.

## 3 Definitions

### 3.1 Definitions of variables

Assume that the item scores are binary manifest variables. Let variable 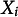 represent the scores (1 = positive, 0 = negative) a random subject obtained on the 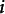 -th item, and denote the full vector of item scores as 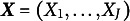 . For each item pair 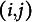 , we assume that there is a discrete variable 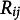 that is used for creating groups in which the conditional covariances are computed. We call the 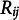 s the conditioning variables, and we assume that they attain integer values ranging from 1 to 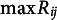 . For example, in Case 2 of Rosenbaum (1984), 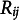 is defined as the sum score on the other items; that is, the items 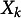 with 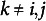 . Then, we have 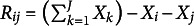 ; we call this the *pairwise rest score*. In Case 5 of Rosenbaum (1984), 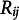 consists of deciles of the pairwise rest score. In the CARP test of Ellis and Sijtsma (2023), 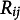 consists of deciles of a weighted sum score on the other items, with weights estimated from a training sample. Let 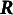 be the vector of all 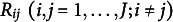 .

### 3.2 The hypothesis

The null hypothesis of interest is

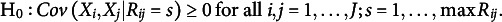



However, the version of the Mantel–Haenszel statistic Rosenbaum (1984) and Ellis and Sijtsma (2023) used for testing 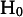 is rather based on a weighted mean of sample covariances, and it would be more precise to say that the null hypothesis is

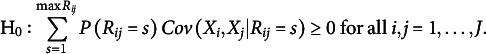



### 3.3 Definition of the pairwise statistics

In this section, we define two sample statistics per item pair 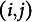 that we aggregate later. First, we define the mean of conditional covariances that estimates the quantity 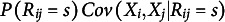 in the null hypothesis. Second, we define the *Z*-statistic, which is the standardized version of the first statistic. The formal definition is the following.

Assume that there are 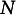 i.i.d. copies of 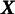 , denoted by 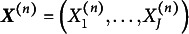 ; 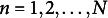 . 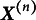 contains the scores of the 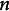 th subject in the sample. Let 
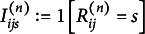
 denote the indicator function for the event 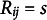 in subject 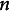 . That is, 
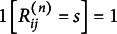
 if 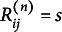 , and 
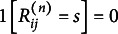
 otherwise. Let 
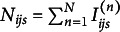
 denote the number of subjects with 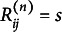 . The conditional covariance in the subgroup with 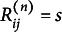 is given by

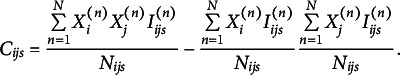



The version of the Mantel–Haenszel statistic Rosenbaum (1984) used is based on standardization of

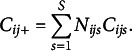



We refer to 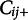 as the MCC. The standardization Rosenbaum used is based on the variance estimate

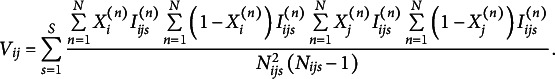



The *Z*-statistic is then defined as

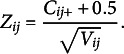



The term 0.5 is a continuity correction.

## 4 Estimation of the covariance matrix of the pairwise test statistics

In this section, we discuss the estimation of the covariance matrix of the 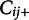 s and the covariance matrix of the 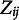 s. These covariance matrices are conceptually like the asymptotic covariance matrices in structural equation modelling (SEM) because they estimate the covariance across all possible samples. However, because the asymptotic covariance matrices in SEM are typically derived from the model and typically pertain to model estimates rather than conditional covariances, we further refrain from focusing on the apparent similarity. Next, we delineate three estimation methods.

### 4.1 Estimation based on sample moments

The equation for MCC contains only sums of products and products of sums, divided by the 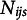 . We worked out a formula for 
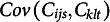
 , assuming that 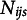 and 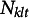 are fixed values rather than random variables (see Appendix). This new equation uses only moments of the variables 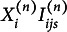 , 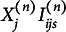 , 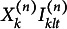 , 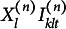 , and their products. By substituting the corresponding sample moments, one obtains an estimate 
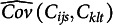
 for 
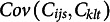
 . Next, the required covariance is estimated as 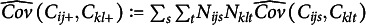 .

It should be noted that in the IRT application of testing the MH model, the 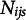 are not fixed. By doing as if the 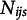 are fixed anyway, we ignore the possibility of a correlation between 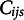 and 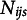 . This might still entail a better approximation than assuming the identity matrix, and we will use the simulation studies to decide whether this approximation is useful.

### 4.2 Estimation based on bootstrapping

#### 4.2.1 Bootstrapping of the MCCs

In this approach, we resample 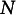 rows of the data matrix with replacement and compute the MCC for each item pair 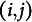 in the resample. Denote the MCC of item pair 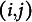 in a resample as 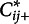 . We resample 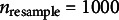 times, thus constructing a matrix of 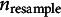 rows and 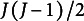 columns, in which each row 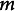 contains the 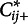 values of the 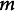 -th resample. Next, we compute the covariance matrix of the 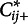 s.

#### 4.2.2 Bootstrapping of the Zs

In this approach, we resample 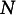 rows of the data matrix with replacement and compute the *Z*-statistic for each item pair 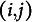 in the resample. Denote the *Z*-value of item pair 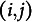 in a resample as 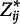 . We resample 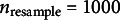 times, thus constructing a matrix of 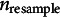 rows and 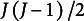 columns, in which each row 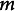 contains the 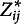 values of the 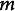 -th resample. Next, we compute the covariance matrix of the 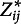 s.

### 4.3 Estimation with the identity matrix

In this approach, the third method, we simply assume that the asymptotic covariance matrix of the 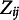 s is the identity matrix. This is comparable to the diagonally weighted least squares (DWLS) method often used for polychoric correlations in ordinal factor analysis (Li, 2015). The reason why we suspect that the identity matrix may work well is that under MH, each conditioning group with a fixed pairwise rest score has only a small remaining variance of the latent variable, which implies that the response variables are close to independent, and independent Bernoulli variables have covariances that are asymptotically uncorrelated (Anderson & Goodman, 1957).

## 5 Aggregation of the pairwise statistics

In this section, we consider the lower-diagonal matrix of 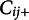 s or 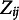 s as a vector 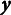 in 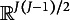 . Let 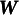 be the covariance matrix of 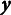 as estimated in any method of the previous section. We consider various methods to aggregate 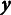 into an omnibus test.

### 5.1 Distance to the nonnegative cone

The theory in this section is based on order restricted statistical inference (Robertson et al., 1988). We project 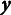 onto the nonnegative cone in 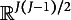 , defined by 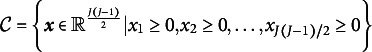 . We define the projection 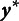 as the point in 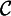 that minimizes the squared Mahalanobis distance 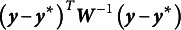 . The chi-bar-square statistic is then defined as this squared distance, i.e.

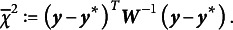



This result serves as the overall test statistic in the decision rules discussed in the next section. The following algorithm was used to obtain 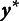 and 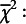

1. Obtain the Cholesky decomposition 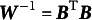 , and let 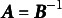 .
2. Compute 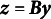 . If 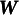 is the covariance matrix of 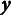 , then the covariance matrix of 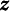 is the identity matrix.
3. Project 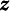 onto the cone 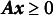 with the function coneA of the R-package **coneproj** (Liao & Meyer, 2014). Let 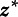 be the result of this projection; then 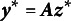 .
4. Compute 
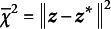
 , where 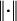 is the Euclidian distance.

### 5.2 Preselected standardized partial sum of pairwise statistics

In their CARP statistics, Ellis and Sijtsma (2023) split the total sample of subjects into a training sample and a test sample. In our treatment hitherto, all statistics were computed with the test sample. However, one can compute 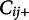 in the training sample as well; denote this as 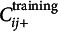 . Let 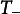 be the set of pairs 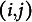 with 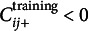 and 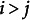 , and denote its size as 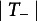 . We add the corresponding values of 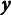 and divide the sum by its standard error. More specifically, let 
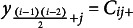
 for all 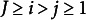 , and define 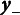 as the subvector of 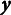 containing the elements 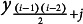 for which 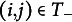 . Let 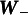 be the submatrix of 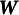 , corresponding to the elements of 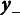 ; Ellis and Sijtsma (2023) defined the omnibus statistic as

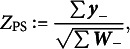

 where 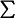 indicates the sum over all elements in the following vector or matrix. Here, we call this omnibus test statistic the preselected standardized partial sum of pairwise statistics. For clarity, if 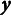 contains the 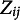 s and 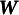 is assumed to be the identity matrix, then

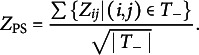



### 5.3 Conditionalized multiple testing procedures

Ellis et al. (2018) argued that multiple testing procedures for interval hypotheses can be enhanced with the following general adaptation: Pick some real number 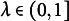 , and select only the p-values with 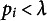 , and divide each 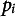 by 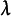 ; then, apply an ordinary multiple testing procedure, such as the Bonferroni correction or the Benjamini-Hochberg correction, to the resulting set. That is, apply an ordinary multiple testing procedure to the set of corrected 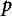 -values 
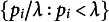
 . Ellis et al. argue that this procedure often controls the Type 1 error, certainly with independent 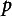 -values, and also with non-independent 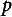 -values if the number of 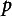 -values is large and the Bonferroni-correction is used. They show that this procedure increases the power if the 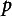 -values are supra-uniform; that is, if most 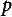 -values are higher than would be expected in a uniform distribution. We expect that the latter condition is often fulfilled in the situation under investigation here, where the 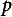 -values test whether conditional covariances are nonnegative in the context of MH. If MH holds and the item response functions are not flat, then the conditional covariances will be positive, yielding supra-uniform 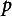 -values.

Ellis et al. (2018) investigated their conditionalization procedure with the multiple testing methods Davidov (2011) discussed in the context of independent 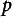 -values, and we will apply some of these multiple testing methods here. Davidov recommended using the 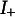 statistics, but the method that he labelled “normal” achieved similar power (Davidov, p. 2439–2440). The latter method means that the 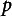 -values are converted to standard normal 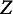 -statistics and added, and this method is the natural candidate if the test statistics underlying the 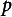 -values are normal. Applied to the present situation with the conditionalization rule of Ellis et al. (2018) and 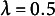 , this amounts to the following. Let 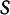 be the set of pairs 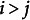 with 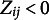 in the test sample, and denote its size as 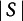 . A test statistic based on the conditionalized sum is

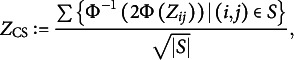

 where 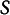 is the number of pairs 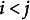 with 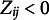 . However, the statistic that was most powerful in the simulations, Ellis et al. reported was the product of 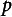 -values, which Davidov attributed to Fisher. In the present situation, after a log-transformation of the conditionalized product, we obtain,

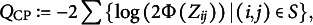

 which is compared to a chi-square distribution with 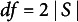 .

Finally, we consider the Bonferroni correction because it is so easy and well-known despite the general consensus that more powerful alternatives exist. In conditionalized form, this amounts to

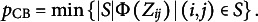



Note that all three statistics, 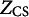 , 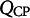 , and 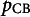 , ignore the correlations of the 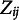 s. In 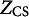 and 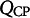 , it is implicitly assumed that the 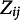 s are uncorrelated, and we are not certain that they control the Type 1 error in correlated cases. However, the correlations between pairwise CARP statistics might be so small that it hardly affect the distribution, as we argued in the section where we proposed the identity matrix. Therefore, we study these statistics despite the uncertainty about possibly correlated 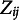 s.

### 5.4 Preselection with multiple testing procedures

The idea of a preselection of item pairs based on the training sample can also be applied to multiple testing procedures, like the conditionalization principle Ellis et al. (2018) discussed. Suppose the data sample is randomly split into a training sample and a test sample. Let the training data matrix be denoted as 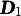 and the test data matrix as 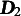 , where 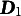 and 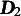 are independent. Suppose that for each subset 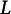 of pairs of variables in 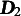 there is a multiple testing procedure 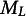 that controls the Type I error when it is applied to 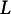 . More precisely, 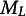 is a function that is applied to 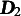 and only uses the pairs of variables in 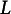 , resulting in a 1 (reject) or 0 (no reject) decision, with 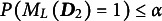 if the null hypothesis is true, where 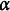 is the nominal level of significance. Suppose that we chose 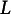 as a function of 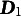 ; let this function be denoted as 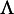 with range 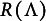 . An example of this would be that 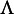 selects the pairs with negative conditional covariances and 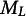 uses the *p*-values 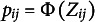 of 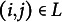 with the Bonferroni correction. That is, 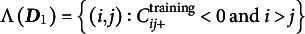 and 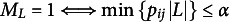 . Then 
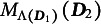
 is the procedure that applies the Bonferroni correction to *p*-values in the test sample using only pairs that have a negative conditional covariance in the training sample. Such procedures control the Type I error rate: Since 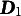 and 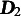 are independent, the conditional distribution of 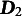 given 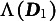 is the same as the unconditional distribution of 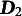 , so the rejection rate is

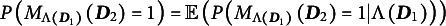




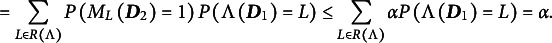



Thus, we may calculate *p*-values for conditional covariances in the test data using only the pairs that have a negative conditional covariance in the training sample and then apply a multiple testing procedure to this selection as if it were the entire test data set from the outset. Applying this procedure to the statistics of the previous section, we obtain the following results. Let 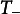 be the set of pairs 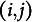 with 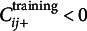 and 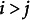 . Then

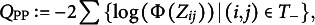

 with 
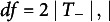
 where 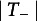 is the size of 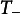 . Similarly,

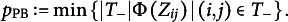



## 6 Decision rules

### 6.1 Decision rules: the LR test and the conditional LR test

In this section, we consider two decision rules based on 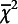 . The first decision rule uses the unconditional distribution of 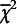 . The second decision rule uses the conditional distribution of 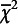 , given the dimensionality of the boundary hyperplane that contains 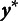 . If 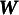 is the identity matrix, then this dimensionality is equal to the number of negative MCCs. Both the 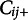 s and the 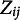 s have an asymptotic multivariate normal distribution as 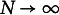 (Browne, 1984, proposition 2). Therefore, we assume a multivariate normal distribution for 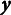 , which is either the vector of 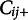 s or the vector of 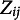 s.

First, we consider the likelihood ratio (LR) test. Using this test, we reject the null hypothesis if 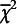 exceeds a critical level (Robertson et al., 1988). Under the least favorable case of the null hypothesis, where 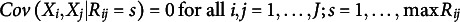 , the distribution of 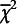 is a weighted average of chi-square distributions (Robertson et al., 1988):

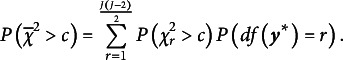



We estimated the weights 
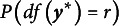
 by drawing from a multivariate normal distribution with a covariance matrix 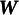 , and counting for each draw how many coordinates are negative. We used 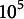 draws. Denote the estimated probability of getting 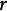 negative coordinates as 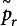 . The 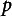 -value for the observed chi-bar-squared is obtained as

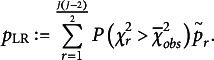



The null hypothesis is rejected if 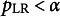 .

Second, we consider the conditional test based on Wollan and Dykstra (1986). Ellis et al. (2018) generalized the conditionalization principle to other multiple testing procedures with one-sided hypotheses and demonstrated that conditionalization achieves a strong gain in power if most null hypotheses are true, a situation that can be expected here. We also discuss other conditional tests; therefore, we call this test the conditional likelihood (CL) ratio test.

Let 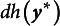 be the dimensionality of the boundary hyperplane on which 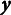 is projected, and let 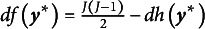 . Wollan and Dykstra explain that the conditional distribution of 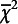 given 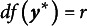 is a chi-square distribution with 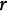 degrees of freedom if 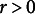 . Let 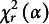 be the right-sided critical value for nominal significance level 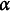 in a chi-square distribution with 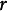 degrees of freedom; that is, 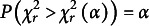 . In the conditional test, we reject the null hypothesis if both 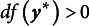 and 
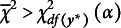
 . Assuming a multivariate normal distribution, the Type 1 error rate of the conditional test is less than 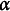 , because, as pointed out by Wollan and Dykstra,

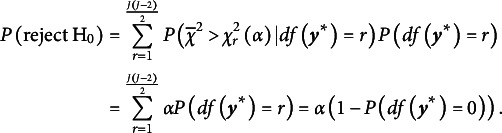



The event 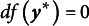 corresponds to 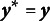 , which would happen if all 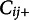 or 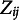 are nonnegative. Wollan and Dykstra continue to estimate this factor, but this probability is small for five items or more, and therefore it is ignored here, consistent with Ellis et al. (2018). In sum, we define

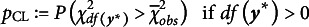




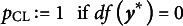



### 6.2 Decision rules: other tests

The p-values of the other tests are computed using

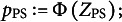




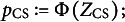




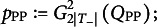




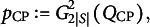

 where 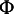 is the standard normal cumulative distribution function and 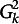 is the chi-square survival function with 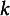 degrees of freedom. The corrected p-values 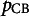 and 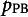 are used without further correction.

## 7 Comparison with competing methods

We compare our statistics with the two prominent alternative methods for the proposed test, which are the DIMTEST or DETECT procedures for analysis of essential dimensionality. DIMTEST and DETECT are two procedures based on Stout’s (1987) theory of essential dimensionality (also Stout et al. 1996). DIMTEST has a confirmative approach that tests unidimensionality, whereas DETECT was created as an explorative approach that divides the set of test items into clusters that are associated with different dimensions. Li et al. (2017, p. 210) summarize DIMTEST as follows:

The DIMTEST procedure (Nandakumar, Yu, Li, & Stout, 1998; Stout, 1987; Stout, Froelich, & Gao, 2001) is often used to test the null hypothesis that an exam is locally independent and unidimensional. It does this by dividing the test into two subtests (an assessment subtest called AT and a partitioning subtest called PT) and testing whether there are any local dependencies among the AT items, conditioned on the score on the partitioning test. DIMTEST has been widely studied for dichotomous item exams and has good power when AT and PT are chosen well (e.g., Froelich & Habing, 2008). If AT and PT are chosen poorly (e.g., both are random samples of items), the procedure will have power near 0.

Like a non-aggregated CARP test, DIMTEST uses conditional covariances, but whereas a CARP test rejects unidimensionality if the conditional covariances are negative, DIMTEST rejects unidimensionality if the conditional covariances are too high. Both CARP tests and DIMTEST divide the set of items into a partitioning test and an assessment test first, but CARP tests restrict the assessment test to a pair of items. Our new aggregated CARP (ACARP) test avoids this problem of selecting an assessment test by aggregating the individual CARP tests across item pairs. DIMTEST handles this problem by splitting the test based on factor analysis in a training sample, and the procedure can be improved further with bootstrapping (Froelich & Habing, 2008). DIMTEST is based on the statistic

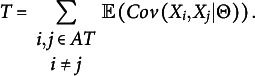



A sample estimate 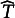 of 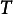 is obtained by replacing 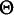 with an estimate 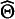 based on the partitioning test, usually the sum score. Stout (1987) argued that 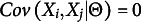 if the test is unidimensional, and, therefore, 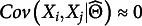 if 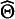 is a good estimate of 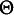 . A high value of 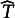 means that the test is not unidimensional. This may be due to multi-dimensionality or lack of local independence. Several adjustments and improvements of DIMTEST have been suggested to estimate or reduce the bias in 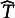 caused by 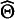 being a fallible estimate of 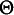 (e.g., Kieftenbeld & Nandakumar, 2015), especially if the number of items is small.

DETECT is a method to cluster items based on their conditional covariances. The clustering is based on Zhang and Stout’s (1999) theory of conditional covariances for tests with a simple structure. The procedure produces a clustering of the items, and a unidimensional test should result in one cluster that contains all items. This is an explorative method, and not a statistical significance test. For a given partition 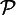 of the set of test items (that is, 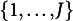 in our notation), the theoretical DETECT index is

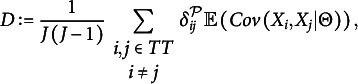

 where 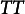 is the set of all items in the test, and 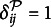 if 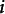 and 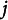 are elements of the same cluster in 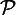 , and 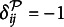 otherwise. DETECT searches for the partition that maximizes 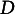 using an estimate 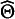 that replaces 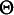 , leading to a sample estimate 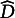 of 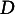 . Many adjustments and improvements of DETECT have been suggested to estimate or reduce the bias in 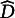 caused by 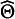 being a fallible estimate of 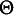 (Roussos & Ozbek, 2006; Zhang, 2007), especially if the number of items is small.

Considering the relationship between DIMTEST and DETECT, we study how DETECT could be used as a confirmatory test of unidimensionality. It would then be logical to define 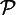 as one cluster that contains all items, and the theoretical DETECT index for this would be

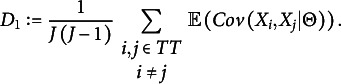



Aside from the factor 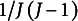 , one could describe 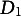 as an instance of 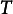 where both the assessment test and the partitioning test contain all items. A high value of 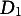 would lead to the conclusion that the test is not unidimensional.

For the sake of comparison, a single CARP test would test whether 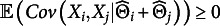 , where 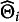 and 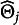 are predictors of 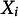 and 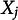 , respectively, based on the items excluding 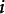 and 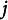 . The 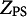 statistic based on the pairwise MCCs 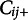 , as defined earlier, can be considered a sample estimate of the theoretical index

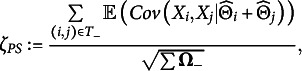

 where 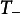 consists of the pairs for which 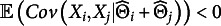 and 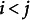 in the training sample, and 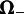 is the covariance matrix used for normalization. A negative value of 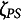 would lead to the conclusion that the test is not unidimensional. The DIMTEST index 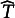 and the DETECT index 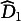 thus have a structure that is very similar to the 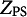 statistic before the latter is normalized with 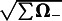 . The main difference is that they are computed using different pairs 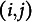 . While DIMTEST would use pairs with *high* conditional covariances in the training sample, 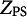 would use pairs with *low* conditional covariances in the training sample. Note that if 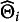 and 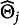 are poor estimates, 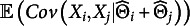 must still be nonnegative, and therefore ACARP does not require bias corrections in order to control the Type I error rate.

DIMTEST, DETECT, and the ACARP tests developed here are closely related. The main difference is the choice of the targeted item pairs and the conditioning variable and the implications that this has for the sign of the covariances. In DIMTEST and DETECT, the conditioning variable is supposed to capture the partitioning test, and unidimensionality is rejected if the conditional covariances in the assessment test are high. In the ACARP tests, one would rather combine covariances of pairs from different dimensions; the conditioning variables are supposed to predict the assessment items, and unidimensionality is rejected if the conditional covariances are negative. For example, if the test has two dimensions 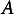 and 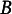 , DIMTEST would use 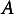 as the assessment test and 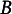 as the partitioning test, or conversely; but 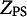 would use pairs 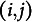 with 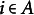 and 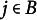 .

## 8 Simulation study I: preliminary selection of test methods

We investigated whether the Type 1 error rate is under control in typical IRT cases, and we compared our test methods on statistical power. In the first simulation study, we aimed to make a preliminary selection of the most promising test methods, which we investigated further in the second and third simulation studies. We used 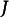 items and a logistic model,




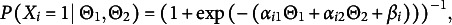



where 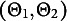 has a bivariate standard normal distribution with correlation 0. Denote the number of items that load on dimensions 1 and 2 as 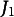 and 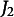 , respectively, so that 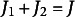 .

For the first simulations, we used 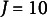 . We studied three possible dimensionality cases:

- Dimensionality 0: In this case, 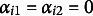 for 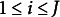

- Dimensionality 1: In this case, 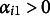 and 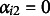 for 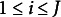

- Dimensionality 2: In this case, 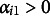 and 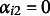 for 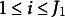 , and 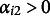 and 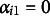 for 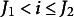 . We used 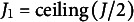 .

The methods discussed allow several ways to obtain a 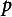 -value: based on 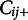 or 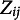 ; aggregated with LR, CL, PS, PP, PB, CS, CP, or CB; and their covariance matrix estimated with the sample moments or bootstrapping or set to the identity matrix. We adopt the following convention to name the tests with four-letter acronyms: The first letter indicates the pairwise statistic (Z for the 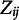 and M for the MCCs, 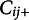 ); the second letter indicates the covariance matrix (B for bootstrapping, M for moments, I for identity matrix, and N for none); the last two letters indicate the aggregation method (LR, CL, PS, PP, PB, CS, CP, or CB). For example, ZICS is based on 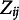 s with the identity matrix and aggregation with the conditionalized sum. An asterisk will be used to indicate a group of tests; for example, ZI** is the group of tests based on the 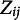 s with the identity matrix. Not all combinations are reasonable: The identity matrix is only reasonable for the 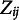 s but not for the 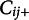 s, and the sample moments and bootstrap method make sense only for LR, CL, and PS. The remaining 20 relevant combinations are displayed in the first column of Table 1. In addition to these tests, we studied the DETECT index 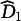 .

**Table 1:** Rejection rates for various tests, based on 1000 samples with fixed item parameters

Pairwise	Estimation covariance	Aggregation	Rejection rate	Rejection rate	Rejection rate
statistic	matrix	method	dimensionality 0	dimensionality 1	dimensionality 2
M	bootstrap	CL	0.074	0.000	0.557
M	bootstrap	LR	0.062	0.000	0.182
M	bootstrap	PS	0.028	0.000	0.596
Z	bootstrap	CL	0.061	0.000	0.529
Z	bootstrap	LR	0.055	0.000	0.168
Z	bootstrap	PS	0.028	0.000	0.592
Z	identity	CL	0.044	0.000	0.554
Z	identity	CP	0.043	0.000	0.584
Z	identity	CS	0.036	0.000	0.587
Z	identity	LR	0.023	0.000	0.336
Z	identity	PP	0.029	0.000	0.755
Z	identity	PS	0.026	0.000	0.686
M	moments	CL	0.042	0.000	0.190
M	moments	LR	0.028	0.000	0.000
M	moments	PS	0.023	0.000	0.312
Z	moments	CL	0.054	0.000	0.349
Z	moments	LR	0.033	0.000	0.000
Z	moments	PS	0.026	0.000	0.368
Z	none	CB	0.027	0.000	0.242
Z	none	PB	0.038	0.000	0.296

*Note:* M = MCC ( 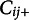 ); Z = pairwise Z-statistic ( 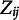 ); LR = likelihood ratio; CL = conditional likelihood ratio; CS = conditional sum; CP = conditional product; CB = conditional Bonferroni; PS = preselected sum; PP = preselected product; and PB = preselected Bonferroni. The item parameters were fixed to 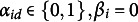 . Each of the 1000 samples contained 1000 subjects.

The simulations were conducted in R, and the code is provided on the Open Science Framework (https://osf.io/hyuzm/). Statistical testing was done at a nominal level of significance 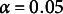 . We programmed the CARP tests with the training sample size equal to 30% of the total sample. For DETECT, we used the confirmatory DETECT function conf.detect of the **sirt** R-package (Robitzsch, 2022), with all items in one cluster; this gives 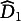 . We rejected unidimensionality if 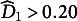 , as recommended in the **sirt** documentation and Roussos and Ozbek (2006, p. 220).

DETECT had a rejection rate of 0 in all circumstances. In the Discussion, we reflect on this result. Tables 1 and 2 show the rejection rates for all other methods. Table 1 shows the rejection rates with all 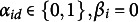 for 1000 samples of 1000 subjects. All 1000 samples in a column are generated with the same parameters. Table 2 shows the rejection rates if the 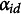 that are not constrained to be zero have distribution 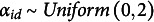 and the 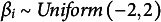 . The 1000 samples in a column of Table 2 all have different parameters, and all contain 1000 subjects.

**Table 2:** Rejection rates for various tests, based on 1000 samples with random item parameters

Pairwise	Estimation covariance	Aggregation	Rejection rate	Rejection rate	Rejection rate
statistic	matrix	method	dimensionality 0	dimensionality 1	dimensionality 2
M	bootstrap	CL	0.055	0.010	0.293
M	bootstrap	LR	0.038	0.000	0.125
M	bootstrap	PS	0.012	0.000	0.232
Z	bootstrap	CL	0.055	0.009	0.288
Z	bootstrap	LR	0.038	0.000	0.123
Z	bootstrap	PS	0.011	0.000	0.217
Z	identity	CL	0.024	0.009	0.289
Z	Identity	CP	0.023	0.008	0.292
Z	identity	CS	0.030	0.010	0.264
Z	identity	LR	0.012	0.000	0.160
Z	identity	PP	0.016	0.003	0.306
Z	identity	PS	0.009	0.000	0.248
M	moments	CL	0.017	0.006	0.065
M	moments	LR	0.008	0.000	0.000
M	moments	PS	0.006	0.000	0.078
Z	moments	CL	0.039	0.021	0.179
Z	moments	LR	0.014	0.000	0.000
Z	moments	PS	0.010	0.000	0.112
Z	none	CB	0.031	0.020	0.168
Z	none	PB	0.025	0.011	0.190

*Note:* M = MCC ( 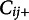 ); Z = pairwise Z-statistic ( 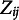 ); LR = likelihood ratio; CL = conditional likelihood ratio; CS = conditionalized sum; CP = conditionalized product; CB = conditionalized Bonferroni; PS = preselected sum; PP = preselected product; and PB = preselected Bonferroni. The item parameters had distribution 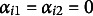 (dimensionality 0), 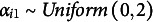 , 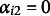 (dimensionality 1), or 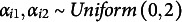 (dimensionality 2), and 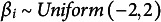 . Each of the 1000 samples contained 1000 subjects.

We conclude that only the following combinations keep the Type 1 error rate under control in both dimensionality 0 and dimensionality 1, at least in the above cases:

- If the covariance matrix is replaced by the identity matrix: all aggregation methods based on the pairwise 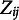 -statistics.
- If the covariance matrix is estimated from sample moments: all aggregation methods based on pairwise 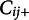 or 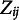 -statistics.
- If the covariance matrix is estimated by bootstrapping: only PS, based on pairwise 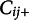 or 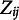 -statistics.

The tests *BCL have a Type I error rate that significantly exceeds 0.05 in Table 1 (*p* = 0.0006). If we omit these tests, and compare the other tests that use a covariance matrix (CL, LR, and PS) across the different versions (M or Z; bootstrap, moments, or identity), then the tests based on the pairwise 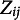 -statistics with the identity matrix have the highest power. The tests where the covariance matrix was based on the sample moments had the lowest power, and we conclude that this method has no advantages.

**Figure 1:**
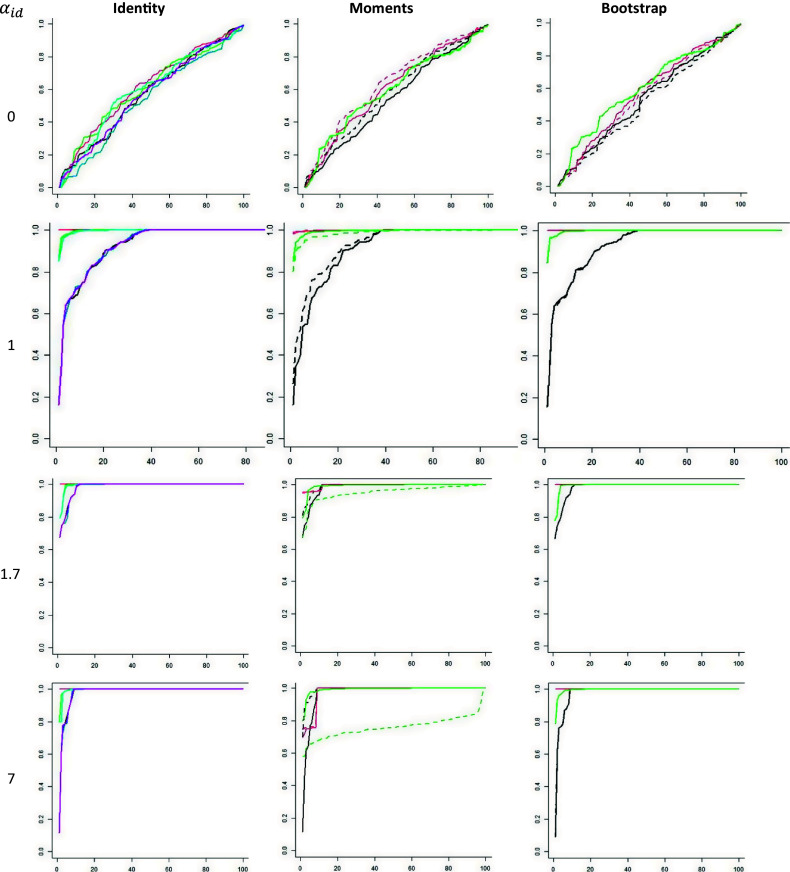
*Plots of p-values showing the Type I error rates.* *Note:* The vertical axis is the *p*-value and the horizontal axis is the rank of the *p*-value. Dashed curve = M***, solid curve = Z***, black = CT, red = LR, green = PS, blue = CS, light blue = PP, and magenta = CP.

**Figure 2:**
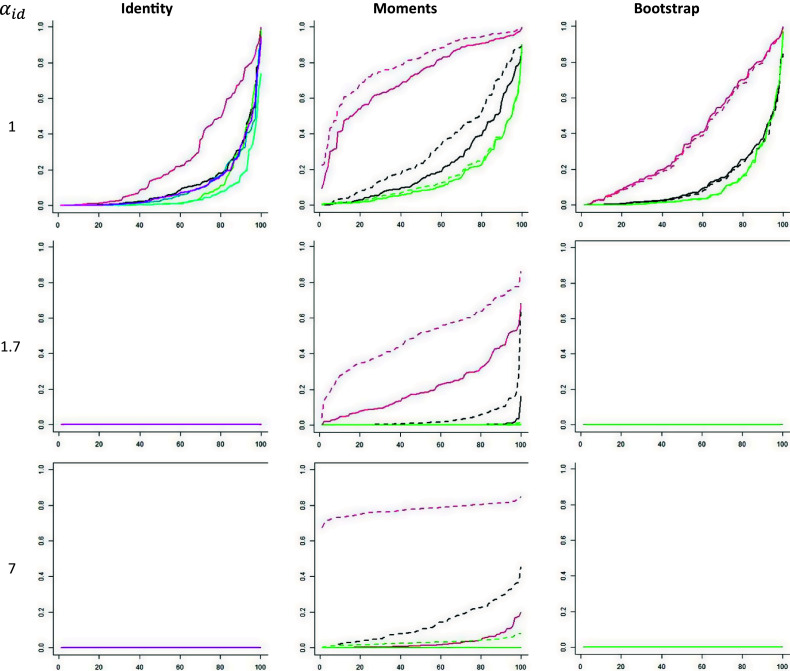
*Plots of p-values showing the power.* *Note:* The vertical axis is the *p*-value and the horizontal axis is the rank of the *p*-value. Dashed curve = M***, solid curve = Z***, black = CT, red = LR, green = PS, blue = CS, light blue = PP, and magenta = CP.

The maximum discrimination parameter 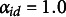 in the simulations of Tables 1 and 2 is rather low. For a broader view, we also conducted simulations with discrimination parameters 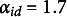 (medium) and 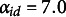 (extremely high) if the item loads on dimension 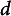 , using 100 simulations of 1000 subjects per case. Figure 1 shows the plots of the *p*-values (Schweder & Spjøtvoll, 1982) for the cases with dimensionality 0 and 1, and Figure 2 shows these plots for dimensionality 2. If the *p*-values have a uniform distribution, they lay on the diagonal line 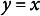 in the plot. These plots confirm the conclusions of Tables 1 and 2: in cases with dimensionality 0 ( 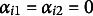 ), all tests produce *p*-values that are approximately uniformly distributed or slightly higher. In cases with dimensionality 1, ( 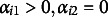 ), all tests produce *p*-values that higher than uniform, and this effect increases with the discrimination parameter. This is to be expected because the population values of the conditional covariances are positive in unidimensional cases with 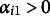 . The power is generally lowest if the covariance matrix is estimated with the moments method. With bootstrapping, each Z***-test produces *p*-values that are very close to the *p*-values of the corresponding M***-test. However, the power of the tests based on the identity matrix matches or outperforms the power of the corresponding tests based on bootstrapping. The next simulation study will therefore focus on the tests based on the identity matrix.

## 9 Simulation study II: comparison of aggregation methods

**Figure 3:**
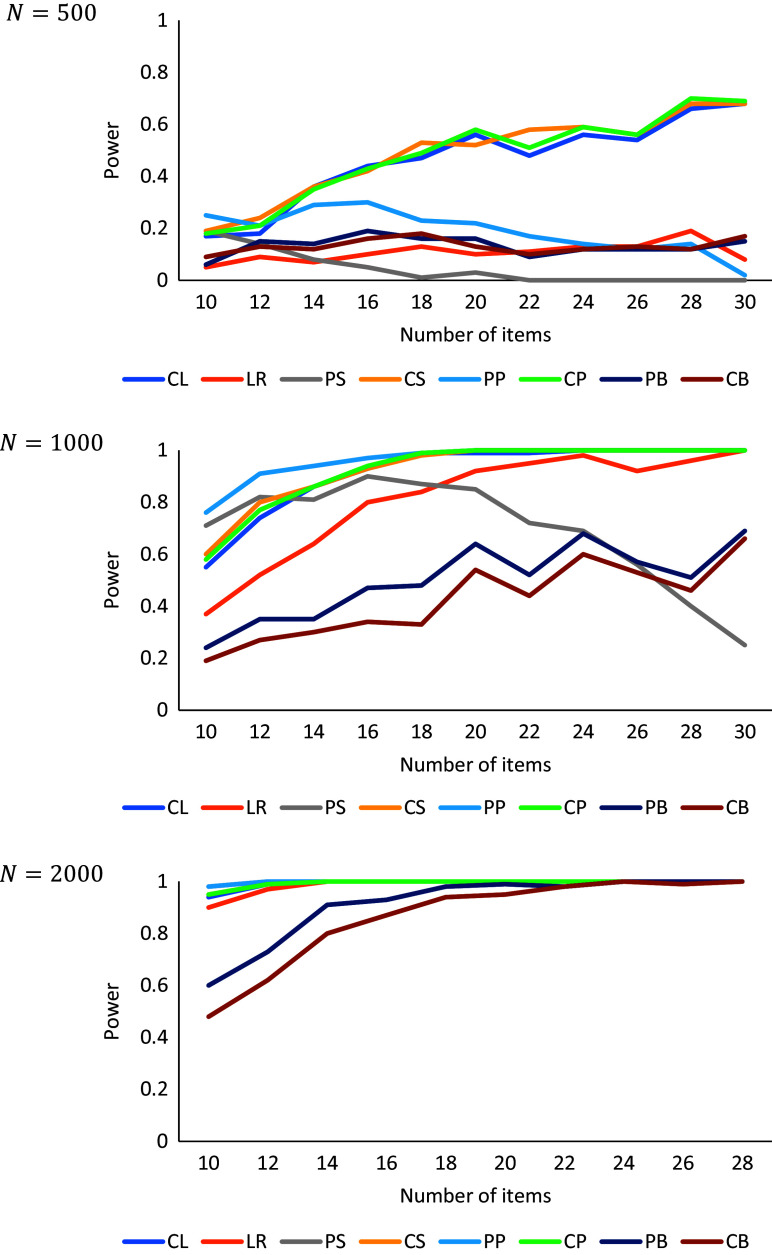
Rejection rates as a function of the number of items and sample sizes.

In this second simulation study, we focussed on the tests that use the identity matrix as the covariance matrix of the 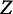 -statistics. The goal was to determine which aggregation methods (CL, LR, PS, CS, PP, CP, PB, and CB) have the highest power and whether this depends on the number of items, number of subjects, and discrimination parameters. The goal was furthermore to determine whether there are cases with unexpected low power. We investigate the effects of the number of items ( 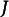 ), sample size ( 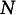 ), and discrimination parameter ( 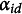 ) on the rejection rates, with fixed item difficulty 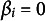 , using 100 simulations per combination. The rejection rates of the two-dimensional cases with low discrimination parameters, 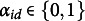 , are shown in Figure 3.

For medium-valued discrimination parameters, 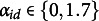 , the estimated power was generally 0.99 or 1.00 even with 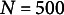 and 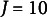 , except for PB and CB. We did not display these excellent power rates in a figure because they do not help discern any pattern. For low discrimination parameters, 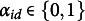 , and 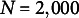 , the power was usually about .90 or higher, with the exception of ZIPB and ZICB. The power differences between the tests are more pronounced for 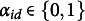 and 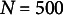 or 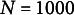 . There we see that the power of ZICL, ZICS, and ZICP increases with the number of items, and that the power of these three tests is generally the highest, except that the power of ZIPP is higher if the number of items is small. The power of ZIPB and ZICB is generally among the lowest and tends to remain low if the number of items increases with 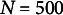 or 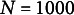 . The power of ZIPS tends to decrease with the number of items if 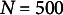 or 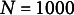 . The power of ZILR tends to remain low if the number of items increases with 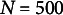 , but slowly increases if 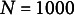 . In sum, the highest power is observed for ZICL, ZICS, ZICP, and sometimes ZIPP. Therefore, we will focus on these tests in the next section.

## 10 Simulation study III: type I error rates

In Simulation Study I, we investigated the Type I error rate only for 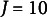 items and a sample size of 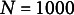 subjects. The present section discusses the Type I error rate more thoroughly, with simulations with varying 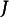 and 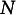 , but only for the ZI** tests, which were selected in Simulation Study I, and we focus especially on the tests ZICL, ZICS, ZICP, and ZIPP, based on their power in Simulation Study II. We label tests with 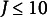 small and tests with 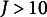 large. Further, we consider 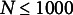 small and 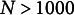 large.

### 10.1 Zero-dimensional cases

We investigated the effects of the number of items ( 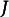 ), sample sizes ( 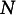 ) on the rejection rates for a nominal significance level of 5%, with fixed discrimination parameters 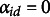 and fixed item difficulties 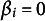 , using 100 simulations per combination. For the number of items, we used all small values 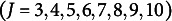 and two large values ( 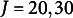 ). For the number of subjects, we used several small values 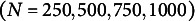 and two large values ( 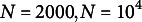 ). For each of the test statistics ZICL, ZICS, ZICP, and ZIPP, the cumulative distribution of rejection counts was larger than the cumulative binomial distribution with 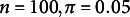 ; that is, the rejection rates were smaller than expected under the binomial distribution. The highest rejection rates (0.07, 0.08, 0.09, and 0.10) were mostly observed with 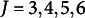 and 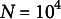 . Therefore a second simulation was conducted with these values of 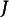 and 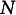 , but with 1000 simulations per combination. The rejection rates for ZICL, ZICS, ZICP, and ZIPP varied between 0.041 and 0.061, and none were significantly greater than 0.05. We also studied this for cases where the 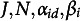 were chosen randomly and independently from uniform distributions with 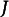 between 3 and 30, 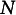 between 250 and 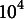 , and 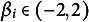 . We sampled 100 cases of 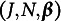 , and generated 100 data sets with a zero-dimensional model for each case. The cumulative distribution of rejection counts was larger than the cumulative binomial distribution with 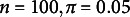 for all ZI** tests except ZIPB. ZIPB had two cases with rejection rates of 0.11. We conclude that the Type I error rate is under control for the tests ZICL, ZICS, ZICP, and ZIPP in these cases.

### 10.2 Unidimensional cases

We investigated the effects of the number of items ( 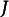 ), sample sizes ( 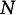 ) on the rejection rates for a nominal significance level of 5%, with fixed discrimination parameters 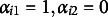 and fixed item difficulties 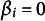 , using 100 simulations per combination. For the number of items, we used all small values 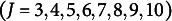 and two large values ( 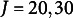 ). For the number of subjects, we used several small values ( 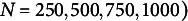 and two large values ( 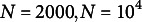 ). The highest rejection rate was 0.01. We also studied this for cases where the 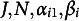 were chosen randomly and independently from uniform distributions with 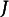 between 3 and 30, 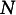 between 250 and 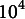 , 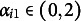 , and 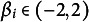 . We sampled 100 cases of 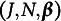 , and generated 100 data sets with a unidimensional model for each case. The rejection rates are given in the first nine rows of Table 3. The rejection rates of ZICP, ZICL, ZICS, and ZIPP were at most 0.04. The cumulative distribution of rejection counts of each of the tests was larger than the cumulative binomial distribution with 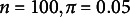 , that is, the rejection rates were smaller than expected under the binomial distribution. Table 3 also shows the rejection rates in various other settings of 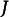 and 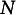 , with similar conclusions.

**Table 3:** *Distribution of rejection rates in various settings of* 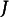 *and* 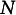 *with unidimensional models*

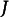		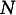										
min	max	min	max	Rejection rate	ZICP	ZICL	ZICS	ZIPP	ZILR	ZIPS	ZICB	ZIPB
3	30	250	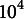	0.00	73	71	70	93	99	93	41	60
				0.01	18	19	22	5	1	4	25	25
				0.02	7	8	8	1	0	2	19	10
				0.03	2	1	0	1	0	1	10	4
				0.04	0	1	0	0	0	0	2	0
				0.05	0	0	0	0	0	0	2	0
				0.06	0	0	0	0	0	0	0	0
				0.07	0	0	0	0	0	0	0	0
				0.08	0	0	0	0	0	0	1	0
				0.09	0	0	0	0	0	0	0	1
10	10	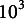	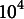	0.00	46	44	47	79	100	92	36	49
				0.01	29	29	24	17	0	6	22	20
				0.02	12	11	16	2	0	0	18	13
				0.03	6	7	9	2	0	2	12	13
				0.04	5	6	3	0	0	0	3	3
				0.05	1	1	0	0	0	0	7	1
				0.06	1	2	1	0	0	0	2	1
10	20	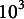	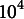	0.00	71	67	65	95	100	96	31	47
				0.01	16	16	25	3	0	2	18	27
				0.02	5	10	6	0	0	2	25	16
				0.03	5	4	2	1	0	0	15	6
				0.04	2	1	1	1	0	0	3	2
				0.05	0	1	0	0	0	0	5	1
				0.06	0	0	0	0	0	0	3	0
				0.07	1	1	1	0	0	0	0	1
3	10	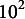	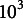	0.00	40	39	43	78	95	82	29	49
				0.01	36	36	32	12	5	11	28	29
				0.02	12	12	12	6	0	5	22	13
				0.03	5	4	7	3	0	2	11	4
				0.04	3	5	3	1	0	0	5	4
				0.05	0	1	0	0	0	0	3	0
				0.06	2	1	2	0	0	0	0	0
				0.07	2	1	1	0	0	0	2	0
				0.08	0	0	0	0	0	0	0	1
				0.09	0	1	0	0	0	0	0	0

*Note:* In each setting of 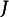 and 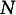 , 100 parameter cases were generated with 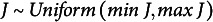 , 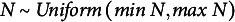 , 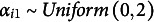 , 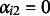 , and 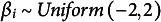 . In each of these 100 parameter cases, 100 samples were simulated. Each cell shows the number of parameter cases with the rejection rate specified in that row. For example, for ZICP, there were 18 cases out of 100 that had a rejection rate of 0.01 over 100 samples in the first setting.

## 11 Simulation study IV: without continuity correction

The previous simulations were conducted with the 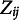 -statistics corrected for continuity with the addition of a term 0.5 in the numerator, as proposed by Rosenbaum (1984) and adopted by Ellis and Sijtsma (2023). Many other continuity corrections exist (Andrés et al., 2024), and we are not sure that a continuity correction is necessary for the sample sizes ordinarily found in IRT. Therefore, we repeated the simulation studies of the ZI-statistics without continuity correction. The simulations of Table 1 and Table 2 are repeated in Table 4 and Table 5 without continuity correction.

**Table 4:** Rejection rates for various tests without continuity correction with fixed item parameters

Pairwise	Covariance	Aggregation	Rejection rate	Rejection rate	Rejection rate
statistic	matrix	method	dimensionality 0	dimensionality 1	dimensionality 2
Z	identity	CL	0.058	0.000	0.657
Z	identity	CP	0.058	0.000	0.686
Z	identity	CS	0.057	0.000	0.684
Z	identity	LR	0.052	0.000	0.447
Z	identity	PP	0.053	0.000	0.835
Z	identity	PS	0.050	0.000	0.756
Z	none	CB	0.039	0.000	0.278
Z	none	PB	0.051	0.000	0.334

*Note:* Z = pairwise Z-statistic ( 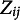 ); LR = likelihood ratio; CL = conditional likelihood ratio; CS = conditional sum; CP = conditional product; CB = conditional Bonferroni; PS = preselected sum; PP = preselected product; and PB = preselected Bonferroni. The item parameters were fixed to 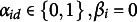 . Each rate is based on 1000 samples of 1000 subjects.

**Table 5:** Rejection rates for various tests without continuity correction with random item parameters

Pairwise	Covariance	Aggregation	Rejection rate	Rejection rate	Rejection rate
statistic	matrix	method	dimensionality 0	dimensionality 1	dimensionality 2
Z	identity	CL	0.045	0.007	0.354
Z	Identity	CP	0.048	0.008	0.361
Z	identity	CS	0.047	0.009	0.332
Z	identity	LR	0.038	0.000	0.212
Z	identity	PP	0.035	0.002	0.381
Z	identity	PS	0.039	0.000	0.300
Z	none	CB	0.035	0.024	0.200
Z	none	PB	0.038	0.013	0.221

*Note:* Z = pairwise Z-statistic ( 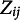 ); LR = likelihood ratio; CL = conditional likelihood ratio; CS = conditionalized sum; CP = conditionalized product; CB = conditionalized Bonferroni; PS = preselected sum; PP = preselected product; and PB = preselected Bonferroni. The item parameters had distribution 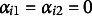 (dimensionality 0), 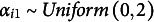 , 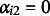 (dimensionality 1), or 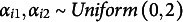 (dimensionality 2), and 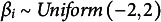 . Each rate is based on 1000 samples of 1000 subjects.

As was to be expected, the rejection rates were now generally larger than in Tables 1 and 2. In Table 4, most Type I error rates (Dimensionality 0) were now 0.05 or slightly higher, and the power (Dimensionality 2) was substantially higher than in Table 1. In Table 5, all Type I error rates are below 0.05, and the power is still larger than in Table 2. The power rates in Tables 4 and 5 are low, but note that these results were obtained for low discrimination parameters ( 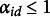 ). We also repeated Simulation Study III without continuity correction, and our conclusion is that the rejection rates were dominated by a binomial distribution with probability 0.05 in all cases, meaning that the Type I error rate is under control. The rejection rates of these versions of ZICL, ZICP, and ZICS are close to 0.05 in the zero-dimensional cases.

Table 6 shows the power rates for the simulations underlying Figure 3, but now repeated without continuity correction, with the positive discrimination parameters set to 1 (low). If the objective is to have power > 0.90, then all four tests achieved this goal with 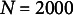 and 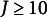 , and also with 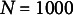 and 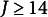 , but not with 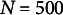 . However, if the positive discrimination parameters are equal to 1.7 (medium), then the power rates were 1.00 even with 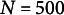 and 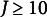 . We did not display these excellent power rates in a table because they were all 1.00.

**Table 6:** Rejection rates for various tests without continuity correction with low discrimination parameters

J	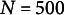				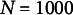				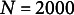			
	CL	CS	PP	CP	CL	CS	PP	CP	CL	CS	PP	CP
10	0.30	0.31	0.37	0.31	0.66	0.77	0.89	0.72	0.95	0.98	1.00	0.96
12	0.35	0.39	0.32	0.37	0.86	0.87	0.93	0.89	1.00	0.99	1.00	1.00
14	0.41	0.43	0.37	0.40	0.92	0.91	0.94	0.92	1.00	1.00	1.00	1.00
16	0.62	0.61	0.51	0.65	0.98	0.98	0.99	0.98	1.00	1.00	1.00	1.00
18	0.61	0.60	0.38	0.64	1.00	0.99	0.99	1.00	1.00	1.00	1.00	1.00
20	0.69	0.64	0.35	0.68	1.00	1.00	1.00	1.00	1.00	1.00	1.00	1.00
22	0.68	0.71	0.33	0.71	1.00	1.00	1.00	1.00	1.00	1.00	1.00	1.00
24	0.74	0.78	0.29	0.81	1.00	1.00	0.99	1.00	1.00	1.00	1.00	1.00
26	0.78	0.74	0.20	0.81	1.00	1.00	1.00	1.00	1.00	1.00	1.00	1.00
28	0.84	0.83	0.23	0.84	1.00	1.00	0.99	1.00	1.00	1.00	1.00	1.00
30	0.81	0.78	0.15	0.82	1.00	1.00	1.00	1.00	1.00	1.00	1.00	1.00

*Note:* Z = pairwise Z-statistic ( 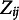 ); CL = conditional likelihood ratio; CS = conditional sum; CP = conditional product; and PP = preselected product. The item parameters were fixed to 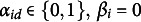 . Each rate is based on 100 samples.

## 12 Conclusions and discussion

We conclude that the pairwise CARP tests Ellis and Sijtsma (2023) proposed can best be aggregated with four of the tests developed here: ZICL, ZICP, ZICS, and ZIPP. These tests control the Type I error rate in a wide variety of test lengths and sample sizes, and their power against two-dimensional alternatives is larger than the power of other aggregate statistics that we studied. ZIPP had the greatest relative power if there were less than 18 items with 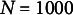 , but not in most other cases. Further investigations are needed to determine whether this is also true for alternatives with more than two dimensions.

The Type I error rates of the ZI-tests are well below the nominal rate of 0.05, and this suggests that improvement is possible. The pairwise *Z*-statistic, as defined by Ellis and Sijtsma (2023), includes a continuity correction that might be too conservative. Based on our simulations, we conclude that the continuity correction may be abandoned for the sample sizes we studied ( 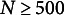 ). Without continuity correction, the Type I error rate is still under control, and the power increases. The Type I error rates of ZICL, ZICP, and ZICS are then close to 0.05 in the zero-dimensional cases of Tables 4 and 5.

The Type I error rate in unidimensional cases is far below 0.05, even without continuity correction, but this does not imply that the tests are too conservative. As an analogy, consider the elementary normal-theory one-sided *Z*-test for a mean 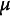 with known variance 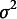 . For the null hypothesis 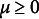 and sample mean 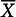 one would use 
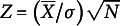
 and reject the null hypothesis if 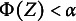 . If real data were generated with 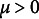 , then 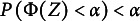 , meaning that the Type I error rate is less than 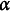 . This is usually not viewed as a sign that something is wrong with the one-sided *Z*-test. The situation in our case is similar because we have a one-sided test for a mean, but in our case it is a mean of conditional covariances. In the unidimensional case, this mean is positive, which reduces the Type I error rate.

If the discrimination parameters equal 1 and the intercepts equal 0, the power rates of ZICL, ZICP, ZICS, and ZIPP are well above 0.90 for 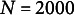 , regardless of whether the continuity correction is used. For these item parameters, if there are at least 14 items and the continuity correction is abandoned, the power is also above 0.90 for 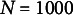 , but the power is substantially below 0.90 for 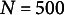 for all studied test lengths between 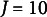 and 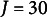 . We emphasize that these power rates were obtained for low discrimination parameters. We consider discrimination parameters of 1 as low because we did not use the general factor 1.7 in our parametrization (unlike e.g., Roussos & Ozbek, 2006). If the positive discrimination parameters equal 1.7 (medium), then the power rates of ZICL, ZICP, ZICS, and ZIPP are 1.00, even with 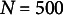 and all investigated test lengths from 10 to 30, based on simulations using 100 samples.

We also compared our statistics with the DETECT index, applied in a confirmatory manner, using the criterion 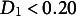 . To our surprise, despite the theoretical similarity of this index to the ZIPS statistic, this index appeared to lack discriminatory power, as it never rejected the hypothesis of unidimensionality. This is a puzzling result, seemingly at odds with the positive evaluations reported by the index’s creators. While we have concerns about the validity of our results for DETECT, we were unable to identify any errors in our code. We believe this issue warrants further investigation.

Our study provides three new statistics for a confirmatory test of unidimensionality in monotone IRT models, and they seem to outperform older methods—at least in the cases we simulated. Still, the power of these methods is somewhat disappointing for sample size 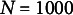 and discrimination parameter 1, and better methods may be possible. A simple improvement might be found in the size of the training sample, which was set at 30% in all our analyses. Furthermore, aggregation of different splits into training samples and test samples might be useful. Finally, it would be worthwhile to investigate which of the four tests can be recommended as most powerful under various circumstances.

## Data Availability

The simulated data and the code that generated it, are available in the Open Science Framework repository at https://osf.io/hyuzm/

## A Appendix

Proposition A1. If  is independent of  then  .
Proof.

### A1 The covariance of two sample covariances

In asymptotic distribution free (ADF) SEM, it is common to obtain the covariance matrix of sample covariances as the sample grows to infinity, which is called the asymptotic covariance matrix (e.g., Browne, 1984). However, it is also possible to obtain an exact formula for the covariance of two sample covariances for finite  , provided that the variables have finite fourth moments.

We develop the covariance of two sample covariances, assuming finite fourth moments of the involved variables. Denote the variables to be studied as  . Suppose that a random sample of  subjects is drawn, and denote the score of subject  on variable  as  , and let  , the score pattern of subject  . We assume that the  subjects are drawn independently, and that therefore their score patterns  are independent, and that each  has the same multivariate distribution as  . Thus, the  are independent copies of  . We study two sample covariances, given by

Using the summation rules for covariances, the covariance of these sample covariances is

We will now develop the four terms in this sum, which will be called the four *main terms*. For the first main term, we need

This sum has  terms, but if  then  , and in the remaining  terms with  ,  . Therefore, we obtain

For the second main term, we need

Similar to the previous term, the outcome of the generic covariance term in this sum,  , depends on which of the indices  are equal. To keep track of this, we constructed Table A1, in which all possible truth values of the equalities  ,  , and  are listed. Each row contains one combination of truth values and the number of terms with that combination. Each row also contains the intermediate expression of the covariance, where the true equalities of that row are substituted in the generic expression  , and the final expression, where this is intermediate expression is rewritten in terms of the central moments of  . A few examples may clarify this:

In the second row, we consider the generic terms  with  . There are  such terms, and substitution of the equality  leads to the intermediate expression  . Since  ,  is independent of  , and therefore this covariance is 0, which is the final expression.

In the third row, we consider the generic terms  with  . There are  such terms, and substitution of the equality  leads to  . Since  ,  is independent of  but not of  . Here, we can use proposition A1, and obtain  .

In the fourth row, we consider the terms with  . This is a logical contradiction, and therefore there are 0 of such terms.

**Table A1:** *Development of the term*

			Count	Intermediate expression	Final expression
0	0	0			
0	0	1			
0	1	0			
0	1	1			
1	0	0			
1	0	1			
1	1	0			
1	1	1			

Summarizing from Table A1, we obtain

For the third main term, we obtain analogously,

For the fourth term, we need to develop

The generic expression for the covariances in this sum is  . There are now four indices,  and therefore there are six equalities that may or may not be true:  All possible truth values of these equalities are listed in Table A2, together with their count, the intermediate expression, and the final expression. For example, in the third row, we consider the case where  , while all other indices are unequal. There are  such terms. Substitution of  leads to  , and then proposition A1 leads to  . In row 13 of Table A2, we used the following reasoning:

A similar argument leads to the final expression in row 19 of Table A2.

**Table A2:** *Development of the term*

							Count	Final expression
1	0	0	0	0	0	0		0
2	0	0	0	0	0	1		0
3	0	0	0	0	1	0		
4	0	0	0	0	1	1		
5	0	0	0	1	0	0		
6	0	0	0	1	0	1		
7	0	0	0	1	1	0		
8	0	0	0	1	1	1		
9	0	0	1	0	0	0		
10	0	0	1	0	0	1		
11	0	0	1	0	1	0		
12	0	0	1	0	1	1		
13	0	0	1	1	0	0		
14	0	0	1	1	0	1		
15	0	0	1	1	1	0		
16	0	0	1	1	1	1		
17	0	1	0	0	0	0		
18	0	1	0	0	0	1		
19	0	1	0	0	1	0		
20	0	1	0	0	1	1		
21	0	1	0	1	0	0		
22	0	1	0	1	0	1		
23	0	1	0	1	1	0		
24	0	1	0	1	1	1		
25	0	1	1	0	0	0		
26	0	1	1	0	0	1		
27	0	1	1	0	1	0		
28	0	1	1	0	1	1		
29	0	1	1	1	0	0		
30	0	1	1	1	0	1		
31	0	1	1	1	1	0		
32	0	1	1	1	1	1		
33	1	0	0	0	0	0		0
34	1	0	0	0	0	1		0
35	1	0	0	0	1	0		
36	1	0	0	0	1	1		
37	1	0	0	1	0	0		
38	1	0	0	1	0	1		
39	1	0	0	1	1	0		
40	1	0	0	1	1	1		
41	1	0	1	0	0	0		
42	1	0	1	0	0	1		
43	1	0	1	0	1	0		
44	1	0	1	0	1	1		
45	1	0	1	1	0	0		
46	1	0	1	1	0	1		
47	1	0	1	1	1	0		
48	1	0	1	1	1	1		
49	1	1	0	0	0	0		
50	1	1	0	0	0	1		
51	1	1	0	0	1	0		
52	1	1	0	0	1	1		
53	1	1	0	1	0	0		
54	1	1	0	1	0	1		
55	1	1	0	1	1	0		
56	1	1	0	1	1	1		
57	1	1	1	0	0	0		
58	1	1	1	0	0	1		
59	1	1	1	0	1	0		
60	1	1	1	0	1	1		
61	1	1	1	1	0	0		
62	1	1	1	1	0	1		
63	1	1	1	1	1	0		
64	1	1	1	1	1	1		

Taking all four main terms together, we obtain:

### A2 The covariance of two sums of conditional covariances with fixed weights

In the test proposed by Rosenbaum’s (1984) case 2, the covariance of two items is considered conditionally on the sum of the other items. The statistics used by Rosenbaum is a standardization of a weighted sum of covariances, where the subgroup sizes act as weights. We will now develop a formula for the covariance of a weighted sum of conditional covariances. Let  be the variable that is used for the conditioning of pair  . In the case of Rosenbaum’s case 2,  would be the sum of the other items, but for our purposes it is sufficient to assume that the range of  is some finite set. The null hypothesis would now be that  for each  in the range of  . Denote the corresponding sample covariance as

Let  be the number of subjects in the subsample with  . Rosenbaum’s statistics is based on standardization of

The goal of this section is to obtain a formula for  . As a first step,

We will now first develop  . In this section, we will assume that  and  are fixed numbers instead of random variables. Then  , so a formula for  would solve the problem. In Rosenbaum’s (1984) application, the  and  are actually random variables, as discussed in the main text.

Let  , the indicator function for the event  in subject  . That is,  if  , and  otherwise, so that  . Then

Therefore  can be developed analogously to the one-sample case of the previous section. We start with rewriting it into four main terms.

Using the results of the previous section, we can express these terms as covariance of the variables  ,  ,  , and  and their products. The first main term is

The second main term is

This is developed in Table A3.

**Table A3:** *Development of the term*

			Count	Intermediate term	Final term
0	0	0			0
0	0	1			0
0	1	0			
0	1	1			
1	0	0			
1	0	1			
1	1	0			
1	1	1			

The third main term is analogously,

The fourth main term is

This is developed in Table A4.

**Table A4:** *Development of*

							Count	Final
1	0	0	0	0	0	0		0
2	0	0	0	0	0	1		0
3	0	0	0	0	1	0		
4	0	0	0	0	1	1		
5	0	0	0	1	0	0		
6	0	0	0	1	0	1		
7	0	0	0	1	1	0		
8	0	0	0	1	1	1		
9	0	0	1	0	0	0		
10	0	0	1	0	0	1		
11	0	0	1	0	1	0		
12	0	0	1	0	1	1		
13	0	0	1	1	0	0		
14	0	0	1	1	0	1		
15	0	0	1	1	1	0		
16	0	0	1	1	1	1		
17	0	1	0	0	0	0		
18	0	1	0	0	0	1		
19	0	1	0	0	1	0		
20	0	1	0	0	1	1		
21	0	1	0	1	0	0		
22	0	1	0	1	0	1		
23	0	1	0	1	1	0		
24	0	1	0	1	1	1		
25	0	1	1	0	0	0		
26	0	1	1	0	0	1		
27	0	1	1	0	1	0		
28	0	1	1	0	1	1		
29	0	1	1	1	0	0		
30	0	1	1	1	0	1		
31	0	1	1	1	1	0		
32	0	1	1	1	1	1		
33	1	0	0	0	0	0		0
34	1	0	0	0	0	1		0
35	1	0	0	0	1	0		
36	1	0	0	0	1	1		
37	1	0	0	1	0	0		
38	1	0	0	1	0	1		
39	1	0	0	1	1	0		
40	1	0	0	1	1	1		
41	1	0	1	0	0	0		
42	1	0	1	0	0	1		
43	1	0	1	0	1	0		
44	1	0	1	0	1	1		
45	1	0	1	1	0	0		
46	1	0	1	1	0	1		
47	1	0	1	1	1	0		
48	1	0	1	1	1	1		
49	1	1	0	0	0	0		
50	1	1	0	0	0	1		
51	1	1	0	0	1	0		
52	1	1	0	0	1	1		
53	1	1	0	1	0	0		
54	1	1	0	1	0	1		
55	1	1	0	1	1	0		
56	1	1	0	1	1	1		
57	1	1	1	0	0	0		
58	1	1	1	0	0	1		
59	1	1	1	0	1	0		
60	1	1	1	0	1	1		
61	1	1	1	1	0	0		
62	1	1	1	1	0	1		
63	1	1	1	1	1	0		
64	1	1	1	1	1	1		

Let us now summarize the results. For arbitrary variables  write

and

Then the result of the previous section can be written as

The result of the present section can be obtained with  ,  ,  ,  :

Thus
